# Healing with animals in the Levant from the 10^th ^to the 18^th ^century

**DOI:** 10.1186/1746-4269-2-11

**Published:** 2006-02-21

**Authors:** Efraim Lev

**Affiliations:** 1 Dep. of Erets Israel Studies and School of Public Health, University of Haifa, Haifa, 31905, Israel

## Abstract

Animals and products derived from different organs of their bodies have constituted part of the inventory of medicinal substances used in various cultures since ancient times. The article reviews the history of healing with animals in the Levant (The Land of Israel and parts of present-day Syria, Lebanon, and Jordan, defined by the Muslims in the Middle Ages as Bilad al-Sham) in the medieval and early Ottoman periods.

Intensive research into the phenomenon of zootherapy in the medieval and early Ottoman Levant has yielded forty-eight substances of animal origin that were used medicinally. The vast majority of these substances were local and relatively easy to obtain. Most of the substances were domestic (honey, wax, silkworm, etc.), others were part of the local wildlife (adder, cuttle fish, flycatcher, firefly, frog, triton, scorpion, etc.), part of the usual medieval household (milk, egg, cheese, lamb, etc.), or parasites (louse, mouse, stinkbug, etc.). Fewer substances were not local but exotic, and therefore rare and expensive (beaver testicles, musk oil, coral, ambergris, etc.).

The range of symptoms that the substances of animal origin were used to treat was extensive and included most of the known diseases and maladies of that era: mainly hemorrhoids, burns, impotence, wounds, and skin, eye, and stomach diseases.

Changes in the moral outlook of modern societies caused the use of several substances of animal origin to cease in the course of history. These include mummy, silkworm, stinkbug, scarabees, snail, scorpion, and triton.

## Introduction

Since ancient times animals, their parts, and their products have constituted part of the inventory of medicinal substances used in various cultures. This phenomenon is marked by both a broad geographical distribution and very deep historical origins. As some authors have shown, animal-based medicines have been utilised since antiquity [1,2].

Testimony to the medical use of animals began to appear with the invention of writing, and is found in archives, papyruses, and other early written historical sources dealing with medicine. Data have been found on such usages in ancient civilisations, such as Egypt and Mesopotamia, which left their mark on the various societies that later arose in the Levant.

Historical sources of ancient Egypt mention the medicinal uses of substances derived from animals, for example, cattle milk, bee honey, lizard blood, ox organs, swallow's liver, bat limbs, ambergris from the sperm whale, and the glands of the musk deer [3-6].

Archives of several civilisations of ancient Mesopotamia, mainly the Assyrian and the Babylonian, contain descriptions of fish oil, beeswax and honey, mongoose blood, turtle shell, goat's skin, gazelle, deer and sheep sinew, bird excrement, and animal fat [7-10].

In ancient China, among many other substances of animal origin, the glands of the musk deer were used [11,12]. In India, the Hindu religion has used five products of the cow for purification since ancient times [13].

Classical medical literature also indicates animals as remedies. In the 5^th^–4^th ^centuries BCE Hippocrates [14] included among many other animal substances the use of cattle milk, chicken's eggs, mammals' horns and sea sponge [15]. About 10% of the substances mentioned in Dioscorides's (1^st ^century CE) *materia medica *[14] were body parts and products of animals [16,17]. Such uses on a smaller scale were common in the Byzantine empire [18].

The Jewish sources, mainly the Mishna (1^st^–3^rd ^centuries CE) and the Talmud (4^th ^– 5^th ^centuries CE), that is, the literature of the Jewish Sages, mention several animals and their medical uses: honey was used to treat bulimia and goat's milk to cure coughing. Snakes, human urine, pearl, mammals' glands, and several other substances were used for different medical conditions [19].

The neo-Aramaic medical tradition, which evolved in the Near East during the Byzantine period, conserving classical medical knowledge, made great medical use of animals [20,21]. This knowledge was conveyed farther and translated, becoming an important part of the new Arab medical and pharmaceutical profession (7^th ^century CE).

Arabic medieval literature offers ample information about animals in general and their medical uses in particular. The 'medicine of the prophets' (*tibb al-nabawi*) indicates intensive medicinal use of chicken eggs, cattle cheese, and bee honey [22,23], for medicinal uses of foodstuff were common in the Middle Ages, as they still are in folk medicine [24]. Early Muslim physicians such as the 9^th^-century al-Tabari [14] and al-Kindi [14] describe the medical uses of several animals, in Iraq and Iran, such as bear, beaver testicles, camel, cattle fat, coral, crab, dog, fish stone, horse, lizard, medical skink, mouse, pearl, pigeon, rabbit, rhino and goat horns, scorpion, snake, squid, turtle, and wolf, and animal products such as honey, wax, milk, and eggs. Together these comprise about 7% of all medicinal substances [25,26]. More information on such uses can be found in general encyclopaedias such as that of the 13^th^-century al-Qazwini [14,27,28]. al-Damiri, the 14^th^-century Muslim zoologist [14], describes in his lexicon hundreds of animals [29], tens of which were used for remedies [30].

The accounts of travellers during the Middle Ages are an additional source of information about animals used for medicinal purposes. For example, Geoffrey of Unseefe (12^th ^century) described the use of theriac against various kinds of insect bites, while Jacques de Vitry (12^th^–13^th ^centuries) describes the beaver, which "bites off its own testicles with its teeth and throws them to the pursuing hunters" who make use of them for medicinal purposes. Jacques de Vitry mentions a pharmacist in Acre who raised various animals and used their excrement to prepare medications. Felix Fabri (15^th ^century) described the hunting of the adder, which provided one of the components for theriac. Thomas Shaw (18^th ^century) tells of the striped lizard found on the coast of Syria and used for the arousal of sexual desire [31].

Many of the animals which were mentioned before are also used in present-day traditional medicine. For instance, in Iraq twelve kinds of animals are described as medicinal sources, including sea sponge, cow, camel, bee, fish, squid, sheep, nacre, and silkworm, and they constitute 5% of all the substances mentioned [32]. A survey conducted in Syria during the 1970s found that 2–8% of the substances in the possession of medicine vendors in the markets were of animal origin [33,34]. A survey of traditional *materia medica *in use in the markets of Israel recorded 20 substances of animal origin [35]. Similar data are derived from surveys conducted in Jordan [36].

In Pakistan, for instance, 31 substances were listed (animal parts and products), constituting 9% of all the medicinal substances in the inventory of traditional medicines. Examination and research show that these substances are similar to those used as remedies throughout human history, irrespective of geographical borders, and include: sea sponge, bee honey and beeswax, squid, medical skink, lizard, silkworm, crab, spider, amber, pearl, nacre, hedgehog, and earthworm [37-39].

A study on the use of medicinal substances in the Levant during the Middle Ages found that 9.5% of all the medicinal substances were of animal origin [40]. The primary purpose of some animal products, which were used as remedies, was food. The study also examined the reciprocal relationship of substances such as milk, cheese, and honey as food products and their use as medicinal remedies [24].

The importance of animal parts in the history of pharmacy in general has been studied since the beginning of the 20^th ^century [41]; other works deal with groups of animals and their uses in medicine, for example, marine animals [42].

## Tools and methods

The present study takes a new approach to the use of animals in medicine in the medieval (10^th^–16^th ^century) and early Ottoman (16^th^–18^th ^century) Levant (the Land of Israel and parts of present-day Syria, Lebanon, and Jordan, defined by the Muslims in the Middle Ages as Bilad al-Sham).

Literature (primary as well as secondary) consisting of tens of diverse historical sources was screened to shed light on the medicinal uses of materials of animal origin from the 10^th ^to the 18^th ^century. The main sources are presented in table 1.

**Table 1:** Main historical sources for the research

**Name**	**Period**	**Description**	**References**
al-Mas'udi	10^th^-century	A Muslim geographer whose travel accounts provide information about production and trade in medicinal materials, including substances of animal origin.	[43]
al-Tamimi	10^th^-century	A Jerusalem physician all of whose works are lost except one, which has recently been studied. Yet his writings are indirectly known through their citation by later authorities such as Maimonides [44, 45] and Ibn al-Baytar [46].	[47, 48]
Genizah Documents	11^th ^century	The Cairo Genizah (depository) contains private and commercial correspondence and legal documents of the medieval Jewish communities of the Eastern Mediterranean. Several 11^th^-century correspondences of Jewish traders contain important information about the trade in and the use of medicinal materials.	[49, 50, 51]
Acre Taxes	13^th ^century	The *Assizes de Jerusalem *contain many documents related to various aspects of the Crusader governing system in the Levant. A list of products traded in Acre, the main commercial city in the Crusader kingdom, and the taxes levied on them was used.	[52]
Benevenutus Grassus	12^th^–13^th ^centuries	Benevenutus was a Frankish ophthalmologist, who in his book on the subject named some of his prescriptions 'Jerusalemics'.	[53, 54, 55]
Jacques de Vitry	12^th^–13^th ^centuries	Bishop of Acre, who published his knowledge accumulated through travelling and reading. His book contains some medical information.	[56, 57]
Rabbi Moshe Ben-Maimon (Maimonides)	12^th^–13^th ^centuries	A Jewish physician and religious philosopher from Andalusia who worked mainly in Egypt, where he was the Sultan's personal physician. Maimonides wrote many medical books.	[44, 45, 58]
Abu Muhammad Abd Allah Ibn Ahmad Ibn al-Baytar	13^th ^century	Andalusian physician and herbalist, who visited the Near East. In *The Compendium of Simple Drugs and Food*, among hundreds of remedies he mentions numerous medicinal substances of animal origin in use in Bilad al-Sham in his day.	[46]
Shams al-Din al-'Uthmani	14^th^-century	A Muslim judge in the Safed region who wrote a description of the city and the surrounding area which contains information about local medicinal substances and their applications.	[59]
Francesco Suriano	15^th^–16^th ^centuries	An Italian trader who became a Franciscan monk, serving his order for many years in the Levant. His unique knowledge was preserved in his *Treatise on the Holy Land*, which contains important information about medieval agriculture and some details about the medicinal substances in use in the Levant at the time.	[60]
Italian Trade	13^th^–15^th ^centuries	Venetian maritime trade documents provide us with information about medicinal materials exported from Acre to Europe by the Venetians. Commercial documents from various archives, mainly Italian, shed light on the trade in spices, agricultural products, and industrial raw materials.	[61]
Frescobaldi	14^th ^century	An Italian traveller who visited the Levant, together with Gucci and Sigoli, in 1384. They published their travel accounts, which contain some information about the medicinal uses of plants and animals.	[62]
Felix Fabri	15^th ^century	A Dominican monk of Swiss origin who visited the Levant. He wrote an important work, with copious information about the region, its residents, their customs, and the goods that were available on the local markets.	[63]
Daud Ibn 'Umar al-Antaki	16^th ^century	A Turkish physician from Antioch who became a well-known writer. His treatise on medicine contains useful information about medieval Islamic medicine and medicinal substances and their usage in the Levant.	[64, 65, 66]
Rabbi Hayyim Vital	16^th^–17^th ^centuries	A Jewish scholar who worked as a physician in Safed, Jerusalem, and Damascus.	[67, 68]
Rafael Mordechai Malki	17^th ^century	An Italian Jewish physician who arrived to Jerusalem in 1677 and became one of the heads of the Jewish community of the holy city and their physician.	[69]
David de Silva	17^th^–18^th ^centuries	A physician and one of the leaders of the Jewish community of Jerusalem. His book *Peri Megadim *supplies us with information about the medicinal uses of the contemporary *materia medica *in Jerusalem.	[70]
Franciscan Lists	18^th ^century	A Franciscan medical institution in Jerusalem was well known in medieval and Ottoman Jerusalem mainly for its rich 'modern' stock of medicinal substances. Few 18^th ^century lists of the medicinal materials in the pharmacy were recently discovered and studied.	[71]

The information presented in Table 2 is the result of a survey of literature on the medicinal substances of Bilad al-Sham (the Levant) from the 10^th ^to the end of the 18^th ^century [40,72]. The criteria were: (1) the animal or product is mentioned in a book on medicine or pharmacy treating the Levant or one of its cities or geographical zones. (2) It is mentioned in a source regarding trading in it in the area; and we possess other historical sources dealing with its medicinal applications in the Levant. (3) The substance is mentioned in general literature regarding its medicinal use.

**Table 2:** Medical substances of animal origin in the Levant in the 10^th^–18^th^century

**Scientific Name**	**Common Name**	**Figure no.**	**Extract/Product**	**Selected Sources**	**Selected Uses**
*Ammoperdix heyi*	Desert partridge		Meat	[70]	Strengthens the stomach
*Angulus *sp.	Sea Shell (Tallina)		Shell	[46]	Mild purgative; women's diseases
*Anser anser*	Goose		Oil	[70]	Unknown
*Apis mellifica*	Honey		Honey	[55, 67, 69, 70]	Skin, eye, and stomach diseases, haemorrhoids, burns and wounds. Strengthens and cleans stomach and lungs.
*Apis mellifica*	Wax	1 – wax	Wax	[49, 55, 70]	Haemorrhoids, burns, and wounds
*Archispirostreptus syriacus*			Body	[67]	Removes unwanted hair from the eyelids
*Avicula margarittifera*	Pearl		Pearl	[70, 71]	Eye, heart, and liver ailments
*Bombyx mori*	Silkworm	2 – cocoon	Cocoon, Larva	[64]	Wounds, throat inflammation, haemorrhoids
*Bos Taurus*	Cow	3 – hard cheese	Milk, Cheese, Butter	[64, 67, 70, 71]	Eye diseases, haemorrhoids, leprosy. Strengthens the stomach, cleans the blood; treats skin diseases
*Capra hircus mambrica*	Goat		Cheese	[67, 70]	Cancer and skin diseases; fattens; enhances libido, reinforces potency
*Castor fiber*	Common Beaver		Testicles	[55, 57, 71]	Eye diseases, animal bites and stings, cramp, epilepsy, hysteria.
*Chamydotis undulate*	Bustard		Body parts	[64]	Eye diseases; breaks up kidney stones
*Cimex lectularius*	Stinkbug		Body	[46, 64, 67]	Clears urinary tract obstructions; jaundice
*Coleoptera *sp.	Scarabees		Body	[67]	Haemorrhoids; enhances libido
*Echis coloratus*	Adder, Ter	4 – adder in the Judean desert	Body	[45, 47, 57, 62, 60, 63, 71]	Basic component of theriac; snake bites
*Equus asinus*	Ass		Body parts	[67]	Haemorrhoids, eye diseases, epilepsy
*Equus asinus *X *Equus cabllus*	Mule		Body parts	[64]	Rheumatism, eye diseases, internal diseases
*Gallus gallus domesticus*	Hen		Egg parts	[67, 70, 71]	Wide variety of uses including reinforcement of potency and enhancing libido
*Gazella *sp.	Gazelle		Horn	[70]	Cleans the blood
*Helix *sp.	Snail		Body	[67]	Haemorrhoids and internal diseases
*Homo sapiens*	Mummy		Mummified body parts	[64, 71]	Headache, skin, internal diseases, ulcer
*Homo sapiens*	Human		Urine	[67]	Sciatica, skin, internal diseases
*Homo sapiens*	Human		Bone	[71]	Unknown
*Homo sapiens*	Human		Stone	[71]	Unknown
*Lampyris *sp.	Firefly		Body	[64]	Breaking up kidney stones; haemorrhoids
*Leiurus quinquestriatus hebraeus*	Scorpion		Body	[67, 71]	Haemorrhoids; skin diseases; component of theriac
*Lumbricus *sp.	Earthworm		Body	[70, 71]	Haemorrhoids; earache, arthritis, clears obstructions of the urinary tract
*Lytta vesicatoria*	Spanish fly		Body	[71]	Raises a blister, counter-irritant
*Merops *sp.	Bee eater		Body parts	[64]	Colds and skin diseases
*Moschus moschiferus*	Musk Deer		Rectal gland	[47, 51, 52, 70, 71]	Purgative; eye diseases, headaches; reinforces potency, heart diseases; 'cold' ailments
*Mus musculus*	House mouse		Ash, body parts	[67]	Haemorrhoids, skin diseases, wounds, insanity
*Muscicapa *sp./*Ficedula *sp.	Flycatcher		Body parts	[64]	Skin, eye, internal diseases; jaundice and spleen inflammation
*Ovis*	Lamb		Body parts	[70]	Strengthens the body, increases weight, cleans the blood
*Paraechinus aethiopicus pectoralis*	Hedgehog		Skin, spines, blood	[67]	Expels fleas (*Pulex irritans*)
*Pediculus *sp.	Louse		Body	[67]	Clears urinary tract obstructions
*Physeter catodon*	Sperm Whale (Ambergris)		Intestinal secretion	[43, 47, 71]	Sore throat, heart diseases, paralysis, cough, cardiac diseases, hysteria
*Rana ridibunda*	Frog		Body	[67, 71]	Haemorrhoids, wounds, bleeding, rheumatism
*Sepia officinalis*	Cuttle Fish	5 – skeleton	Skeleton	[46, 71]	Skin and tooth diseases; clears obstruction of the urinary tract
*Struthio camelus*	Ostrich egg shell		Egg shell	[67]	Eye diseases
*Titurus vittatus*	Triton		Body	[46, 59, 64]	Reinforces potency and enhances libido
*Tubipora Musica *or *Corallium rubrum*	Coral		Body	[50, 61, 70, 71]	Eye diseases and bleeding; strengthens the heart; headache, cough rheumatism, kills worms
Unidentified	Ant		Body	[67]	Jaundice
Unidentified	Lizard		Secretion	[67]	Eye diseases
Unidentified	Kermes		Insect body	[71]	Unknown
Unidentified	Fish		Meat	[70, 71]	Helps digestion, treats internal diseases, strengthens the nerves
Unidentified	Lacca		Secretion	[71]	Unknown
*Vivera civetta*	Civat Cat (Zebed)		Gland secretion	[67]	Reinforces male potency; ear inflammation
*Vulpes *sp.	Fox		Oil	[71]	Unknown

Several animals and their products were disregarded since they were used by quacks or for mystical medicinal uses: these are subjects for different research, although the two fields are not always fully distinguished [67].

## Results

Finding the substances of animal origin in the literature was tough work, but identifying them according to their medieval names, written in many dialects, or by their nicknames was even harder, mainly because no drawings or paintings were available. Therefore, exact scientific identification was not always accomplished.

The means of identification included all old and new dictionaries, lexicons, zoology books, and scientific keys.

The sources noted in the table are those in which the substance is said to be used in the Levant. However, the medicinal uses of the substances in the medieval period were collected from other sources as well, mainly medical books of the same period [27,28,46,64,73,74]. The information presented in the table is limited, so conflicting identifications, detailed descriptions, a full list of sources, or a complete list of uses are not given.

## Discussion and conclusion

The accumulated data set out in table 2 attest to a remarkably wide range of medicinal uses of animals and their parts in the medieval and Ottoman Levant. Recent historical surveys located and identified 286 medicinal materials, of which 81.8% are of plant origin, 5.2% of minerals, 3.5% of other materials, and 9.5% of animal origin [40,75]. Recent ethnopharmacological surveys conducted at shops and with vendors of traditional medicine in Israel [35] and Jordan [36] reveal that similar materials are in use in the same geographical area until present day.

Tables 2 contains forty-eight animal extracts and products. These can be divided into three different groups:

### 1. Available animal substances

Apparently, the great majority of the substances were readily available to the medieval and early Ottoman physician, pharmacist, or patient as wild animals (ant, bee eater, cuttle fish [image no. 5], desert partridge, earthworm, fish, firefly, flycatcher, fox, frog, gazelle, hedgehog, lizard, scarabees, scorpion, sea shell (tallina), snail), as domesticated animals (ass, cattle [cheese – image no. 3], hen, honey and wax [image no. 1], goat, goose, lamb, mule, silkworm [image no. 2]), or as parasites of humans or domesticated animals (louse, mouse, stinkbug). The use of these substances could be explained by their abundance, which ensured fresh and cheap availability of supply.

**Figure 1:**
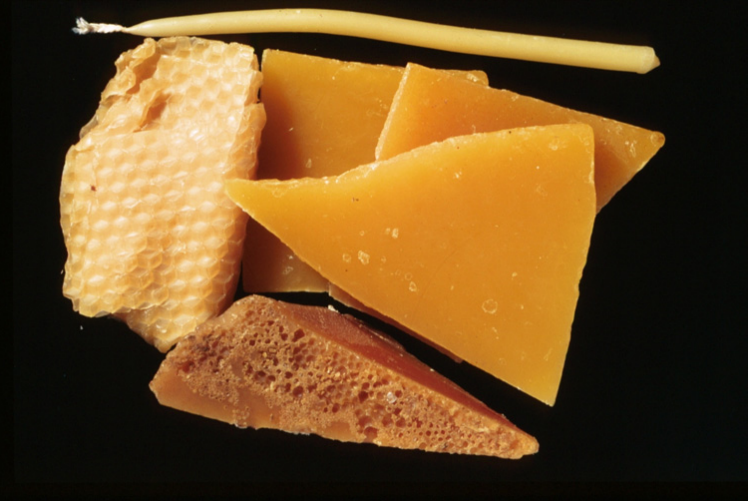
Wax (*Apis mellifica*).

**Figure 2:**
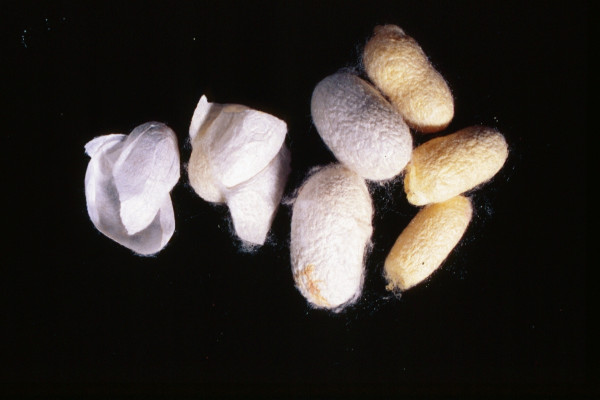
Silkworm Cocoons (*Bombyx mori*).

**Figure 3:**
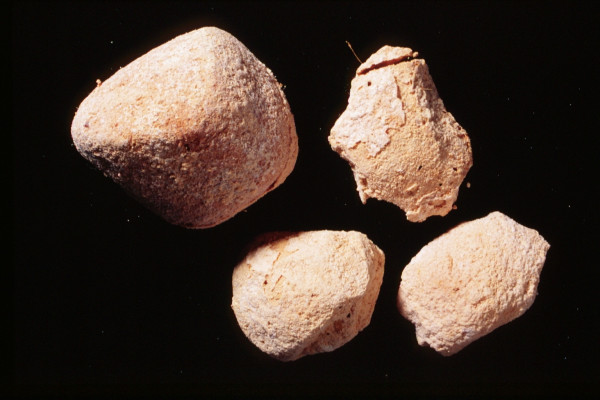
Hard cattle cheese (*Bos Taurus*).

**Figure 5:**
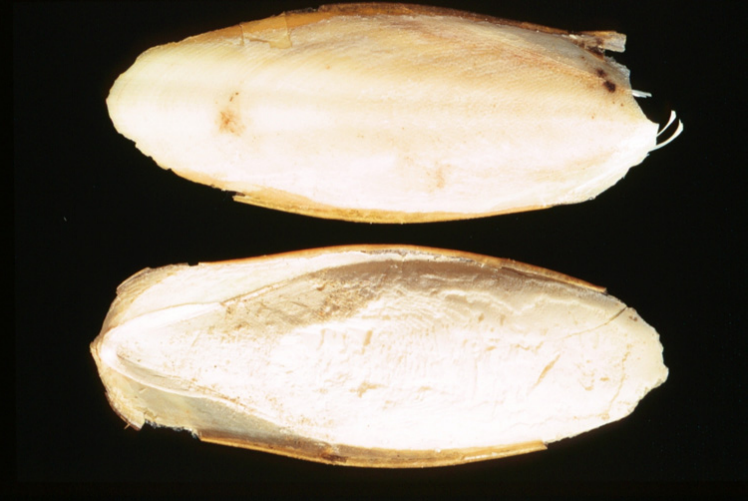
Cuttle fish's skeleton (*Sepia officinalis*).

### 2. Rare animal substances

Adder [image no. 4], bustard, coral, kermes, lacca, ostrich, triton and squid. These animals were hunted in season or collected in the desert, on the seashore, or in remote areas. They were rare, relatively expensive, but reasonably available.

**Figure 4:**
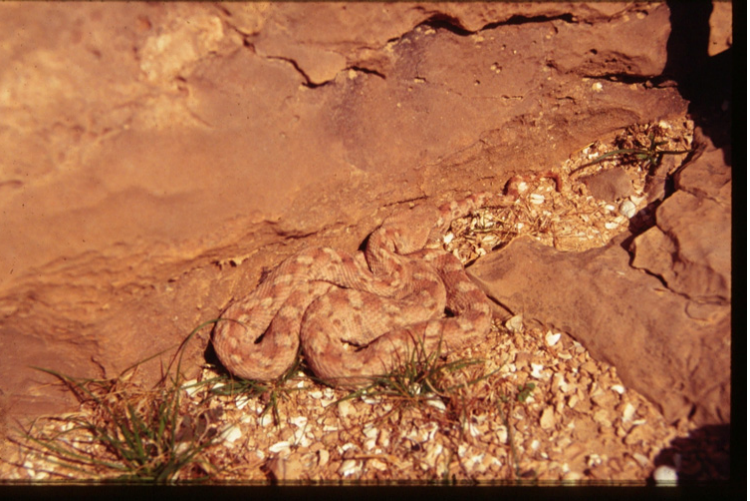
Adder (*Echis coloratus*), taken at the Judean Desert.

### 3. Exotic animal substances

Common beaver, civet cat (zebed), musk, pearl, Spanish fly, sperm whale (ambergris), which were imported from distant lands via the trade routes and therefore were exotic [76]. The habitats of these animals were special and found in distant places. They were very rare, and trade in them was usually a monopoly. They were very expensive.

The animal substances were applied to treat a wide range of symptoms and diseases such as skin diseases, bleeding, wounds, internal disease, haemorrhoids, animal bites, and sex-related diseases. Some of the substances listed in table 2 were used for other purposes such as perfume (musk oil, ambergris), lighting (wax), and food (honey, cheese, chicken products, goat products).

It is assumed that in the medieval and early Ottoman Levant a larger number of animals served for medical use. Presumably, animals such as pigeons and leeches were used but are not mentioned in the sources since they were associated with magic healing or traditional medicine. Some medieval and Ottoman sources do mention these animals, but without any direct connection to the Levant.

Besides the animals and their products that were positively identified, several indications of the medicinal uses of other animal-related materials were detected.

These materials are presented here separately owing to their uniqueness:

I. Petrified spines of sea urchin (*Cidaris sp*.). These were used to open obstructions in the renal system and dissolve renal stones (bladder as well as kidney stones) [77]. It seems as these specific uses were according to the doctrine of signatures [78]. Other uses of this substance were for treating stings, bites, and wounds, and for softening hard skin [46,64,69]. This substance is presented here on account of its animal origin before the petrifaction process. During the medieval and early Ottoman period it was considered as substance of mineral origin, as it still is in present-day traditional medicine in the Levant.

II. 'Theriak', (theriac). This is a mixture of many medicinal substances: plants, poisonous minerals, and extracts of animals generally poisonous such as snakes and scorpions. Its preparation was considered a unique medical and pharmaceutical art [79]. It was used in medieval and early Ottoman Levant to treat leprosy, snakes bites, scorpion stings, animal bites, and poisoning by different kinds of poisons [40]. It was one of the special goods exported from the Holy Land during this period [80].

At times, the use of certain substances of animal origin was against the patient's or the physician's religious precepts (e.g., the internal use of snakes, louse, mummy and scorpions by Jews). Accordingly, Muslim physicians used wine as a medicinal substance even for internal use! The explanation of this phenomenon may be that the medical tradition (mainly classical) that predominated throughout ages superseded local religious norms, especially in cases of saving life or in other hazardous situations.

From a commercial point of view, we readily observe that some of the substances of animal origin were the usual goods traded in the Levant and in international commerce throughout history. Most of the imported products, such as musk, mummy, beaver, coral, and ambergris, were brought from Asia and Africa by sea and land. The majority of them were goods in transit at the Levantine cities and ports, having been sold to western traders, primarily Italians, who shipped them on to Europe. Some substances of local animal origin, such as the triton and the adder, were exported, according to the historical sources, to Egypt and other Mediterranean countries [76,80].

Out of all the historical sources that have contributed information on the use of substances of animal origin in the Levant, two should be mentioned: **Daud Ibn 'Umar al-Antaki**, the 16^th ^century Turkish physician [64-66], and **Rabbi Hayyim Vital **[67,68]. Each of these contributed data on the medicinal uses of nine animal substances, and both lived and practised medicine at the 16^th^–17^th ^centuries – a period in which the medieval medical knowledge reached its zenith in the Levant.

The use of several materials of animal origin came to a halt in the course of history owing to a change in the moral outlook of modern societies. These materials include mummy, silkworm, goat products, stinkbug, scarabees, snail, scorpion, and triton [81].
